# Associations of Bilateral Deficit during Jumping with Physical Performance in Tennis Players

**DOI:** 10.5114/jhk/196460

**Published:** 2025-07-21

**Authors:** Linjie Shu, Jiacheng Zhang, Tingting Chen, Hui Dong, Zhen Wang, Jiancai Chen, Min Hu, Jingwen Liao

**Affiliations:** 1 Guangdong Provincial Key Laboratory of Physical Activity and Health Promotion, Guangzhou Sport University, Guangzhou, China.; 2 Department of Physical Education, Guangdong University of Finance, Guangzhou, China.

**Keywords:** racquet sports, change of direction, linear sprint

## Abstract

This study aimed to investigate the associations of bilateral deficit (BLD) during jumping with physical performance represented by linear sprints and changes of direction (CODs) in tennis players. Thirty-eight tennis players (10 females and 28 males) completed a test program that included three types of jumping tests to determine BLD, two types of linear sprint tests (10 m and 20 m) to assess sprint performance, and three types of COD tests (505COD, 5105COD, and T-test) to evaluate COD performance. The three types of jumping tests included countermovement jumps (CMJs), squat jumps (SJs), and horizontal countermovement jumps (HCMJs). The bilateral index (BI) in jump height (JH), flight time (FT), peak force (PF), peak power (PP), average power (AP), and the force impulse (FI) were then calculated to quantify BLD. Change of direction deficit (CODD) was calculated by subtracting linear sprint test time from COD test time. Results showed that the BI during the CMJ was positively correlated with the linear sprint index (1 correlation, r = 0.33, p = 0.045) and most COD indices (19 correlations, r = 0.32–0.40, p = 0.013–0.049). The BI during the SJ was positively correlated with linear sprint indices (2 correlations, r = 0.32–0.35, p = 0.033–0.049) and most COD indices (17 correlations, r = 0.32–0.44, p = 0.006–0.049). The BI during the HCMJ was positively correlated with COD indices (12 correlations, r = 0.32–0.41, p = 0.010–0.049). Conclusively, BLD during jumping is positively associated with linear sprint and COD performance in tennis players, indicating that lower limb BLD could serve as an index to optimize physical performance and training.

## Introduction

As a sport with nearly 90 million global participants (Haggerty, 2021), tennis remains a sport where training and evaluation methods have significant potential to develop. Tennis is a multidirectional sport that features numerous unilateral movement patterns (Fernandez-Fernandez et al., 2009). Sufficiently developed physical performance, such as accelerations, decelerations, and changes of directions (CODs), is the foundation for tennis players to win matches (Pluim et al., 2023). During matches, both linear sprint and COD performance are critical to the gait patterns and stroke quality (Baiget et al., 2019), since a tennis player runs an average of 3 m per stroke and changes the direction four times per minute (Fernandez et al., 2006). COD performance, in particular, has a moderate to high impact on overall tennis performance (Volk et al., 2023). Additionally, there is a positive correlation between jump height (JH) during the countermovement jump (CMJ) and peak serve speed (Hayes et al., 2021), further highlighting the importance of explosive lower limb strength in tennis. In addition, the high volume of unilateral movements and specific technical demands contribute to the nature of asymmetry in tennis (Madruga-Parera et al., 2020), which, however, may be associated with physical performance (Coratella et al., 2018) and sports injury (Bishop et al., 2023). This complexity highlights the importance of exploring lower limb asymmetry and its associations with different physical performance in tennis, thereby optimizing the training prescription and enhancing competitive fitness in tennis players.

The growing interest in bilateral deficit (BLD) highlight its potential application in sport training, where the total force produced by maximal unilateral contractions surpasses that of maximal bilateral contractions (Škarabot et al., 2016). BLD shows high plasticity, with unilateral resistance training tending to promote BLD, whereas bilateral resistance training might inhibit it (Gonzalo-Skok et al., 2017). Since tennis is characterized by various movement patterns, the previous study investigated BLD during a single type of jumping (CMJ) in tennis players, and showed that those with higher BLD in JH and a force impulse (FI) achieved better COD performance (Kozinc & Šarabon, 2021). Thus, it is also necessary to investigate BLD during jumping of different contraction types and movement directions among tennis players. Notably, sprinting ability is beneficial for tennis performance in net interception, creating offensive opportunities, and returning deep range shots at baseline. Even though no statistical association of BLD during jumping with sprint performance was reported among healthy university students (Bishop et al., 2021) and young soccer players (Ascenzi et al., 2020), volleyball players with higher BLD in JH (during CMJs) presented better sprint performance (Pleša et al., 2022). Thus, the association of lower limb BLD with linear sprint performance still requires exploration.

Therefore, this study incorporated the CMJ, the squat jump (SJ), and the horizontal countermovement jump (HCMJ) for BLD assessment to address these research gaps to investigate the associations of BLD during jumping with linear sprint and COD performance in national second-class tennis players. Due to the plasticity of BLD and numerous unilateral movement patterns in tennis, we hypothesized that BLD might serve as an index to monitor tennis training if it is beneficial for physical performance in tennis players.

## Methods

### Participants

Thirty-eight tennis players (10 females and 28 males) were recruited for this study; their characteristics are described in Table 1. Inclusion criteria we applied were as follows: national second-class tennis players, ≥ 5 years’ experience of tennis training, ≥ 8 hours of tennis training per week, and no severe musculoskeletal injuries for at least one year. Tennis players volunteered to participate in the study in June 2023. All participants were informed about the procedures of the study and signed informed consent. The experiment was approved by the Human Experimental Ethics Inspection of the Guangzhou Sport University (protocol code: 2023LCLL-54; approval date: 22 September 2023) and was performed in accordance with the principles of the Declaration of Helsinki.

**Table 1:** Descriptive characteristics and sex differences of participants in this study.

Variable	Total	Female	Male	*p*
No. of participants	38	10	28	
Age (years)	22.76 ± 2.09	22.50 ± 2.80	22.86 ± 1.82	0.961
Body height (m)	1.74 ± 0.07	1.66 ± 0.06	1.76 ± 0.05	<0.001
Body mass (kg)	68.45 ± 9.73	60.26 ± 9.27	71.37 ± 8.21	0.008
BMI (kg/m^2^)	22.64 ± 2.33	21.78 ± 2.30	22.95 ± 2.31	0.503
**Training experience**				
Years of training (years)	8.66 ± 2.41	8.30 ± 2.41	8.64 ± 2.45	0.987
Weekly training (hs)	12.79 ± 4.31	12.50 ± 6.04	12.89 ± 3.64	0.368

Data are represented as mean ± SD; BMI = body mass index; p < 0.05 indicates significant differences between females and males

### Study Design

**Figure 1:**
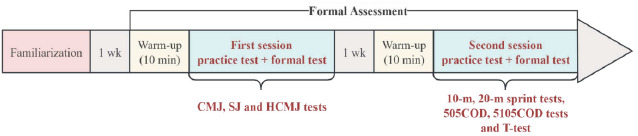
Overview of the assessment procedure.

This was an observational study. The assessment procedures were conducted as shown in Figure 1. One week before the formal assessment, all participants underwent a familiarization session with the assessment protocol at testing sites. The formal assessment consisted of two sessions. The first session evaluated BLD through three types of jumping tests (CMJ, SJ and HCMJ) under both bilateral and unilateral conditions, and the second session determined linear sprint (10 m and 20 m) and COD (505COD test, 5105COD test, and T-test) performance one week after the first session. During each session, participants began with a 10-min warm-up (5-min jogging and 5-min bodyweight resistance exercises); passive stretching was avoided for impacting the outcomes of physical performance (Knudson et al., 2001). Afterwards, 2–3 practice attempts (at 50–75% of each participant’s maximal effort) for each test were performed, and then the formal test was conducted with two trials. All variables were recorded and calculated as the average values of the two trials for subsequent analysis (Meylan et al., 2010b).

### Jumping Assessment

During formal jumping assessment, each participant completed CMJ, SJ, and HCMJ tests in sequence. For each type of jumping, the maximal unilateral jump tests and bilateral jump tests were performed in randomized order. Participants were allowed a rest interval of ≥ 30 s between repetitions of the same test, and the interval time between two different types of jumping tests was ≥ 2 min. During all jumping tests, participants placed hands on the hips at all time. During unilateral jumping tests, the tested lower limb was responsible for balancing and jumping, and the other lower limb was not allowed to touch the tested side or to swing during jumping. A professional test-taker gave encouragement and rectification to standardize the jumping movements of participants. Any deviation from test protocols of the CMJ, the SJ or the HCMJ necessitated retaking the test after one minute (Bishop et al., 2021; Kozinc et al., 2022).

#### Countermovement Jump Test

The vertical CMJ test has been used to evaluate tennis players’ lower limb neuromuscular capability (Tufano et al., 2018). During unilateral and bilateral CMJ tests, participants were instructed to squat down to a self-selected depth, and then to jump as high as possible with full lower-limb extension.

#### Squat Jump Test

During unilateral and bilateral SJ tests, participants were instructed to squat down until the knee angle reached 90°, then to hold this position for 2–3 s, and to jump as high as possible with full lower-limb extension. To ensure the standardization of the SJ tests, the force-time curves were carefully checked by the test-taker. If the curve dropped down significantly before jumping, the test was void and then retaken after one minute (Kozinc et al., 2022).

#### Horizontal Countermovement Jump Test

During unilateral and bilateral HCMJ tests, participants stood on the starting point, then jumped as far as possible, and remained stable when landing, as no additional hops were allowed. During unilateral HCMJ tests, participants jumped with the tested lower limb as far as possible and landed with both lower limbs stably with no additional hops (Ascenzi et al., 2020).

### Bilateral Index Calculation

Lower limb BLD assessments were performed on a triaxial force plate (AMTI, USA), and the force data were recorded at a sampling rate of 1000 Hz. Mathematical software (Matlab, USA) was utilized to calculate JH (during CMJ and SJ), jump distance (JD, during HCMJ), flight time (FT), peak force (PF), peak power (PP), average power (AP) and the FI during jump tests, according to the protocols in previous studies (Meylan et al., 2010a, 2010b). The outcomes of BLD (represented by the bilateral index, BI) were calculated using the following formula:


$$\text{BI}\left(\%\right)=\left(\frac{Bilateral}{Right\;leg+Left\;leg}-1\right)\times 100\%$$

A negative BI (%) value indicated a bilateral deficit, and a positive BI (%) value indicated bilateral facilitation (Anders et al., 2021; Železnik et al., 2022).

### Linear Sprint Performance

**Figure 2:**
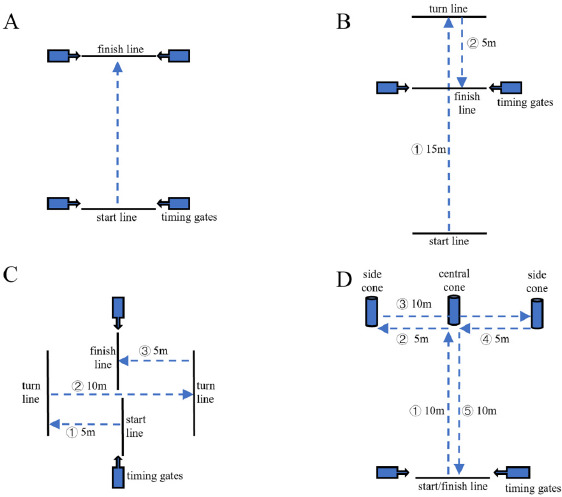
Schematic representation of linear sprint and change of direction (COD) tests. (A) Linear sprint (5 m and 10 m) test; (B) 505 COD test; (C) 5105 COD test; (D) T-Test.

Sprinting demands both high acceleration and high velocity (Dwyer and Gabbett, 2012). Considering tennis’s specificity, the length of linear sprint tests should be no more than 20 m (Kovacs, 2006). Schematic representation of linear sprint tests is shown in Figure 2A. Beam laser timing gates (Brower, USA) were utilized to assess the time of the linear sprint (10 m and 20 m). Timing gates were placed at the 1-m height from the ground, and 30 cm apart from the start line to avoid miscontact. Participants prepared in a static position behind the start line. Participants were free to choose when to start sprinting and then to accelerate as quickly as possible until they passed the timing gates on the final line. All tests were repeated twice with a rest interval ≥ 2 min in between. The interval time between different types of linear sprint tests was ≥ 5 min (C. Bishop et al., 2023).

#### Change of Direction Performance

The ability of COD encompasses the skills of accelerating, decelerating, and changing directions efficiently (Schneider et al., 2023). The assessment of COD performance can provide instructions to enhance agility in tennis players (Cooke et al., 2011). Participants completed three types of tests: 505COD, 5105COD, and the T-test, as shown in Figure 2. The test repetition was set as mentioned above. Moreover, COD deficit (CODD) was calculated through adjusting the outcomes of COD tests via sprint time (T): 505CODD = 505COD time − T_10 m_; 5105CODD = 5105COD time − T_20 m_ (Hernández-Davó et al., 2021).

#### 505 Change of Direction

The timing gates were placed at 30 m from the start line. Participants prepared in a self-conditioned manner behind the start line and were free to choose when to start executing procedures shown in Figure 2B, i.e., sprint forwards 15 m to cross the turn line with one leg, and then sprint forwards 5 m to pass the timing gates at the finish line.

#### 5105 Change of Direction

The timing gates were placed at 10 m from the start line. Participants prepared with one-hand on the ground and ran in the direction of the hand touching. Figure 2C shows the procedures when the left hand touched the ground: first, sprint 5 m to cross the line and turn at 180°; then sprint 10 m to cross the other line and turn at 180°; finally, sprint back 5 m to cross the timing gates at the finish line.

#### T-test

The T-test has been widely used in agility assessments (Jansen et al., 2021). The timing gates were placed at 30 cm from the start line. The central cone was set up at 10 m from the timing gates, with two more cones placed on either side of the central one at 5 m. Participants prepared in a self-conditioned manner behind the start line and were free to choose when to start executing procedures as shown in Figure 2D: first, sprint forwards 10 m to touch the central cone with one hand; then side shuffle 5 m to touch the first side cone; next side shuffle 10 m to touch the other side cone; afterwards, side shuffle 5 m to touch the central cone; and finally, run backwards 10 m to cross the timing gates at the finish line (Pereira et al., 2018).

### Statistical Analysis

All the data were collected using Excel 2021 (Microsoft, USA). Statistical analysis was performed using SPSS 26.0 (SPSS, USA). Descriptive statistics are presented as means ± standard deviations (SDs). Reliability of two repetitions was evaluated using intraclass correlation coefficients (ICCs) (Koo and Li, 2016) and the coefficient of variation (CV = (typical error/mean)×100%). The reliability of measurements was considered acceptable when ICC > 0.75 and CV < 10% (Hopkins, 2000). Correlations of the BI during jumping with linear sprinting and COD performance were assessed using the Pearson’s correlation coefficient (r), and interpreted as negligible (< 0.1), weak (0.1–0.4), moderate (0.4–0.7), strong (0.7–0.9), and very strong (> 0.9). Statistical significance was set at *p* < 0.05.

## Results

### Linear Sprint and Change of Direction Performance

Descriptive statistics for linear sprint and COD performance are summarized in Table 2. Generally, males exhibited significantly better performance in linear sprint indices (10 m and 20 m), and most COD indices (505COD-L, 505COD-R, 5105COD-L, 5105COD-R, T-test-L, and T-test-R) compared to females (*p* < 0.05). Most tests showed relative reliability and acceptable absolute reliability, except for 505CODD-L and 505CODD-R.

**Table 2:** Descriptive characteristics of linear sprint and change of direction (COD) performance among study participants.

Variable	Total	Reliability		Female	Male	*p*
		ICC	CV			
**Linear Sprint (s)**						
10 m	1.92 ± 0.13	0.89	2.16%	2.06 ± 0.13	1.87 ± 0.09	<0.001
20 m	3.30 ± 0.24	0.80	1.74%	3.61 ± 0.22	3.19 ± 0.14	<0.001
**COD (s)**						
505COD-L	2.61 ± 0.19	0.95	4.18%	2.76 ± 0.19	2.56 ± 0.16	0.003
505CODD-L	0.69 ± 0.14	0.71	15.74%	0.7 ± 0.12	0.69 ± 0.15	0.898
505COD-R	2.59 ± 0.20	0.82	3.23%	2.74 ± 0.19	2.54 ± 0.18	0.005
505CODD-R	0.68 ± 0.14	0.68	12.41%	0.68 ± 0.12	0.67 ± 0.14	0.963
5105COD-L	5.58 ± 0.39	0.79	1.95%	5.97 ± 0.41	5.43 ± 0.27	<0.001
5105CODD-L	2.27 ± 0.23	0.92	4.78%	2.37 ± 0.27	2.24 ± 0.20	0.128
5105COD-R	5.56 ± 0.39	0.77	2.91%	5.93 ± 0.39	5.43 ± 0.30	<0.001
5105CODD-R	2.26 ± 0.24	0.84	7.16%	2.32 ± 0.25	2.24 ± 0.24	0.346
T-test-L	12.53 ± 1.45	0.96	1.92%	13.71 ± 1.43	12.1 ± 1.22	0.001
T-test-R	12.42 ± 1.48	0.93	3.00%	13.71 ± 1.65	11.96 ± 1.13	0.002

Data are represented as mean ± SD; ICC = intraclass correlation coefficient; CV = correlation of variation; 505CODD = 505COD deficit; 5105CODD =5105COD deficit; L = left; R = right; p < 0.05 indicates significant differences between females and males

### Bilateral Index during Jumping

**Figure 3:**
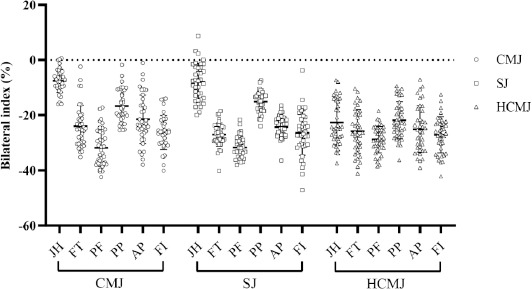
Bilateral deficit index for countermovement jump (CMJ), squat jump (SJ), and horizontal countermovement jump (HCMJ) variables. BI = bilateral index; JH = jump height; FT = flight time; PF = peak force; PP = peak power; AP = average power; FI = force impulse

All descriptive statistics and BI outcomes for jump performance are represented in Table 3 and Figure 3. Almost all participants exhibited negative BI values (−1.28 to −47.20) indicating BLD, except for three participants in JH during the CMJ and four participants in JH during the SJ (bilateral facilitation). The lowest BI was observed in peak force (PF) during the CMJ (−31.84 ± 6.74), the SJ (−31.72 ± 3.99) and the HCMJ (−28.69 ± 4.86), while the highest BI was found in JH during the CMJ (−7.98 ± 8.41) and the SJ (−8.01 ± 6.75), as well as PP during the HCMJ (−21.88 ± 6.65). Most indices showed relative reliability, except for FT during the unilateral (left) CMJ.

**Table 3:** Descriptive statistics during jumping and bilateral index (BI) outcomes among study participants.

	CMJ	SJ	HCMJ
**Bilateral**			
JH# (m)	0.31 ± 0.05	0.29 ± 0.05	1.06 ± 0.16
FT (s)	0.50 ± 0.06	0.49 ± 0.05	0.40 ± 0.04
PF (N/kg)	23.42 ± 2.65	24.40± 2.49	7.74 ± 0.75
PP (W/kg)	47.46 ± 6.19	48.51 ± 6.52	16.07 ± 3.10
AP (W/kg)	3.95 ± 0.84	9.16 ± 1.83	3.03 ± 0.71
FI (Ns/kg)	11.77 ± 1.12	10.31 ± 1.26	3.06 ± 0.45
**Left**			
JH# (m)	0.17 ± 0.03	0.16 ± 0.03	0.68 ± 0.13
FT (s)	0.33 ± 0.05	0.33 ± 0.04	0.27 ± 0.04
PF (N/kg)	17.25 ± 1.57	17.86 ± 2.23	5.50 ± 0.73
PP (W/kg)	28.56 ± 4.26	28.69 ± 4.71	10.35 ± 2.41
AP (W/kg)	2.48 ± 0.51	6.10 ± 1.38	2.01 ± 0.54
FI (Ns/kg)	7.86 ± 0.92	7.02 ± 0.91	2.17 ± 0.28
**Right**			
JH# (m)	0.17 ± 0.03	0.16 ± 0.03	0.69 ± 0.11
FT (s)	0.33 ± 0.05	0.34 ± 0.04	0.27 ± 0.04
PF (N/kg)	17.20 ± 1.72	18.00 ± 2.29	5.42 ± 0.72
PP (W/kg)	28.60 ± 4.43	28.72 ± 4.55	10.40 ± 2.25
AP (W/kg)	2.58 ± 0.66	6.06 ± 1.34	2.04 ± 0.49
FI (Ns/kg)	8.16 ± 0.96	6.95 ± 1.04	2.03 ± 0.38
**BI (%)**			
JH#	−7.98 ± 8.41	−8.01 ± 6.75	−21.75 ± 7.35
FT	−23.99 ± 7.57	−27.03 ± 4.23	−25.58 ± 7.68
PF	−31.84 ± 6.74	−31.72 ± 3.99	−28.69 ± 4.86
PP	−16.55 ± 5.40	−15.11 ± 3.83	−21.88 ± 6.65
AP	−21.55 ± 8.79	−24.27 ± 3.94	−24.81 ± 8.38
FI	−26.27 ± 6.21	−25.72 ± 8.45	−26.90 ± 6.68

Data are presented as mean ± SD; CMJ = countermovement jump; SJ = squat jump; HCMJ = horizontal countermovement jump; JH# indicates jump height for both CMJ and SJ, and signifies jump distance for HCMJ; FT = flight time; PF = peak force; PP = peak power; AP = average power; FI = force impulse

### Correlations of BLD with the Physical Performance Index

Correlations of BLD (represented by the BI) during jumping tests with linear sprint and COD indices are summarized in Table 4.

**Table 4:** Correlations of the bilateral index (BI) during the countermovement jump (CMJ), the squat jump (SJ) and the horizontal countermovement jump (HCMJ) with linear sprint and change of direction (COD) indices.

Variables	JH#	FT	PF	PP	AP	FI
**BI during CMJ**						
10-m sprint	0.33*	0.06	0.07	0.23	0.12	0.26
20-m sprint	0.30	0.15	0.17	0.32	0.12	0.27
505COD-L	0.30	0.27	0.32*	0.31	0.36*	0.34*
505CODD-L	0.10	0.32	0.37*	0.20	0.38*	0.22
505COD-R	0.36*	0.12	0.16	0.35*	0.31	0.24
505CODD-R	0.21	0.12	0.17	0.29	0.34*	0.10
5105COD-L	0.31	0.26	0.27	0.40*	0.28	0.32*
5105CODD-L	0.21	0.28	0.28	0.35*	0.37*	0.26
5105COD-R	0.37*	0.24	0.32	0.40*	0.27	0.27
5105CODD-R	0.29	0.24	0.35*	0.32	0.32*	0.15
T-test-L	0.27	0.27	0.29	0.34*	0.24	0.38*
T-test-R	0.33*	0.21	0.23	0.31	0.30	0.36*
**BI during SJ**						
10-m sprint	0.25	0.24	0.35*	0.26	0.26	0.19
20-m sprint	0.26	0.27	0.32	0.32*	0.25	0.16
505COD-L	0.33*	0.04	0.37*	0.31	0.32*	0.11
505CODD-L	0.22	−0.18	0.18	0.19	0.20	−0.03
505COD-R	0.44**	0.02	0.24	0.23	0.27	0.13
505CODD-R	0.40*	−0.21	0.01	0.09	0.15	0
5105COD-L	0.32*	0.15	0.29	0.42**	0.35*	0.23
5105CODD-L	0.27	−0.04	0.16	0.39*	0.33*	0.23
5105COD-R	0.29	0.17	0.28	0.38*	0.31	0.24
5105CODD-R	0.19	−0.01	0.12	0.28	0.25	0.22
T-test-L	0.39*	0.24	0.33*	0.25	0.25	0.36*
T-test-R	0.36*	0.33*	0.30	0.19	0.19	0.34*
**BI during HCMJ**						
10-m sprint	0.05	0.15	0.20	0.21	0.27	0.14
20-m sprint	0.13	0.18	0.22	0.22	0.26	0.17
505COD-L	0.12	0.10	0.40*	0.37*	0.41*	0.27
505CODD-L	0.12	−0.01	0.36*	0.3	0.31	0.24
505COD-R	0.15	0.12	0.20	0.36*	0.15	0.18
505CODD-R	0.17	0.03	0.09	0.32	−0.04	0.12
5105COD-L	0.24	0.17	0.32	0.23	0.30	0.32
5105CODD-L	0.28	0.09	0.32*	0.15	0.23	0.37*
5105COD-R	0.22	0.12	0.28	0.23	0.25	0.29
5105CODD-R	0.22	0.01	0.23	0.15	0.14	0.30
T-test-L	0.25	0.28	0.31	0.32*	0.37*	0.38*
T-test-R	0.21	0.29	0.29	0.30	0.37*	0.36*

Data are presented as r (Pearson’s correlation coefficient); JH# indicates jump height for both the CMJ and the SJ, and signifies jump distance for the HCMJ; FT = flight time; PF = peak force; PP = peak power; AP = average power; FI = force impulse; JD = jump distance; 505CODD = 505COD deficit; 5105CODD =5-10-5 change of direction deficit; L = left; R = right; * p < 0.05; ** p <0.01

During CMJ tests, the BI in JH showed a positive weak correlation with the 10-m sprint index (r = 0.33, *p* < 0.05). Moreover, the BI in JH, PF, PP, AP, and FI showed weak correlations with 505COD indices (7 correlations, r = 0.32–0.37, *p* < 0.05), and weak to moderate correlations with 5105COD indices (8 correlations, r = 0.32–0.40, *p* < 0.05). Additionally, the BI in JH, PP, and FI exhibited weak correlations with T-test indices (4 correlations, r = 0.33–0.38, *p* < 0.05).

During SJ tests, positive and weak correlations were found between the BI in PF and the 10-m sprint index (r = 0.35, *p* < 0.05), and between the BI in PP and the 20-m sprint index (r = 0.33, *p* < 0.05). In addition, the BI in JH, PF, and AP showed weak to moderate correlations with 505COD indices (5 correlations, r = 0.32–0.44, *p* < 0.05), particularly with the 505COD-R index (r = 0.44, *p* < 0.01). Besides, the BI in JH, PF, and AP showed weak to moderate correlations with 5105COD indices (6 correlations, r = 0.32–0.42, *p* < 0.05). Furthermore, the BI in JH, FT, PF, and FI exhibited weak correlations with T-test indices (6 correlations, r = 0.34–0.39, *p* < 0.05).

During HCMJ tests, no correlation was found between the BI and linear sprint indices (r = 0.05–0.28, *p* > 0.05). However, the BI in PF, PP and AP showed weak to moderate correlations with 505COD indices (5 correlations, r = 0.36–0.41, *p* < 0.05). Also, the BI in PF and FI showed weak correlations with 5105CODD-L indices (2 correlations, r = 0.32–0.37, *p* < 0.05). Additionally, BI in PP, AP, and FI exhibited weak correlations with T-test indices (5 correlations, r = 0.32–0.38, *p* < 0.05).

## Discussion

Abundant evidence is increasingly focusing on the study of BLD in sports, but there are little data to specify the associations between BLD and physical performance among tennis players. Therefore, we focused on tennis players with significant training experience, investigating BLD during three types of jumping (performed with different contraction types and movement directions). The current findings demonstrated that BLD during vertical jumps (CMJs and SJs) was in positive correlations with linear sprint and COD performance, suggesting that BLD during vertical jumps (CMJs and SJs) may serve as an index to monitor linear sprint and COD performance for tennis players. In addition, BLD during the HCMJ was also positively correlated with COD performance, but not with sprint performance, which indicates that higher BLD during horizontal jumps (HCMJs) might contribute to better COD performance, which is of high importance for tennis training. Thus, lower limb BLD may present substantial implications for optimizing performance and athletic training in tennis players.

The earliest study on the associations between BLD and sprint performance was reported in 2010 (Bračič et al., 2010), showing that elite sprinters with higher BLD might produce a lower total FI. However, the current study showed that most BLD during the CMJ was not correlated with linear sprint performance except for BLD in JH with better 10-m sprint performance in tennis players. Interestingly, JH during the unilateral CMJ was found to be correlated with linear sprint performance in basketball players (Barrera-Domínguez et al., 2024), highlighting that this relationship may be context-specific and dependent on sport demands. This could be explained by the cyclic movement and non-combativeness nature of the specific sport, especially since a positive correlation of BLD in JH during the CMJ with linear sprint performance has been reported in volleyball players (Pleša et al., 2022), and no significant relationship was also noted in healthy male university students (Bishop et al., 2021). The CMJ is widely performed during tennis-specific movements, such as the serve, the forehand, and the backhand, which can be utilized to evaluate the ability to rapidly generate force during the stretch-shortening cycle in the vertical direction. The present study indicates that higher BLD is advantageous for COD performance among tennis players. These associations of BLD during the CMJ with COD performance are consistent with previous literature considering basketball, tennis (Kozinc and Šarabon, 2021), volleyball (Pleša et al., 2022), and healthy university students (Bishop et al., 2021). The current study also shows that tennis players with higher BLD performed better in the T-test, which is inconsistent with available data on volleyball players (Pleša et al., 2022). This discrepancy could be attributed to COD practice during routine training among tennis players, while volleyball is characterized by the high prevalence of explosive jumping. Additionally, as most tennis strokes occur from the baseline of the court, lateral movement is predominant during tennis training and matches (Pereira et al., 2018), which corresponds to the T-test movement.

In addition to the CMJ, the SJ test (another vertical jump test) was conducted to assess the ability to rapidly develop force during purely concentric contraction. We found that tennis players with higher BLD exhibited better performance of linear sprints. As for sports other than asymmetric, individualized or net sports, BLD during the SJ was not significantly associated with linear sprint performance in physically active males (Nicholson and Masini, 2021) and elite youth soccer players (Ascenzi et al., 2020). Furthermore, the present study indicated a beneficial effect of BLD during the SJ on COD performance among tennis players. The concentric contractions during the SJ are frequently performed in quadriceps when tennis players start a stroke from the preparation stance. Therefore, developing higher BLD during the SJ might be advantageous for improving both linear sprint and COD performance.

Jumping in the horizontal direction is another critical component for tennis, thus this study further evaluated the associations of BLD during the HCMJ (characterized by the horizontal direction) with physical performance in tennis players. There was neither significant correlation of BLD during the HCMJ with linear sprint performance nor of BLD (in JD and FT) with COD performance in tennis players. Similar results were also reported in elite youth soccer players (Ascenzi et al., 2020). However, the present study also indicated that higher BLD in other indices (PF, PP, AP, and FI) was advantageous for COD performance. Since jumping forward and changes in directions are both prevalent in tennis (approach shot, overhead smash, diving volley etc.), it is reasonable to state that BLD during the horizontal jump is correlated with COD performance in tennis players.

Collectively, the current study showed that during vertical jumps, BLD in JH was most correlated with COD performance among tennis players. Indeed, the JH index comprehensively reflects the neuromuscular strength of lower limbs and is easily accessible for practitioners to evaluate BLD during vertical jumps to monitor tennis training. Moreover, BLD in PP showed the most correlation with COD performance, indicating that BLD represented by explosive force in lower limbs may serve as an essential index to assess the sprint and agility in tennis players.

Unilateral resistance training is better for athletes to improve unilateral strength (Zhao et al., 2023), develop COD performance (Fisher and Wallin, 2014), and enhance the coordination stability (Bogdanis et al., 2019) than bilateral resistance training. Due to the high level of plasticity in BLD, we recommend that when practitioners aim to improve linear sprint and COD performance in tennis players, they might consider increasing the proportion of unilateral resistance training. Practitioners can incorporate both unilateral and bilateral exercises into the training regimen to promote physical performance in tennis players.

It is important to note some limitations within this study. Firstly, most participants involved in the present study were males, thus the conclusions may not be comprehensive in both sexes. Secondly, although the linear sprint and COD performance comprise an important part in tennis performance, tennis specific tests were not applied. In this way, the associations of BLD with tennis-specific performance should be further explored.

## Conclusions

Higher BLD during horizontal jumping results in better COD performance, while higher BLD during vertical jumping is associated with both linear sprint and COD performance among tennis players. Thus, monitoring lower limb BLD could be valuable in optimizing tennis physical performance and training. Since unilateral resistance training tends to promote BLD while bilateral resistance training might reduce it, we recommend that when practitioners aim to improve linear sprint and COD performance in tennis players, they might consider increasing the proportion of unilateral resistance training. For instance, single-leg squats and single-leg jumps are beneficial to improve explosive power and coordination of unilateral lower limbs. Besides, single-leg bridge exercise helps strengthen the glutes and the lower back, which would assist in improving performance of horizontal movements in tennis. Practitioners may incorporate both unilateral and bilateral exercises into their training regimens to promote physical performance in tennis players.
